# Favorable spring conditions can buffer the impact of winter carryover effects on a key breeding decision in an Arctic‐breeding seabird

**DOI:** 10.1002/ece3.8588

**Published:** 2022-02-09

**Authors:** Rolanda J. Steenweg, Glenn T. Crossin, Holly L. Hennin, H. Grant Gilchrist, Oliver P. Love

**Affiliations:** ^1^ 3688 Department of Biology Dalhousie University Halifax Nova Scotia Canada; ^2^ Environment and Climate Change Canada National Wildlife Research Centre Carleton University Ottawa Ontario Canada; ^3^ 113680 Department of Integrative Biology University of Windsor Windsor Ontario Canada

**Keywords:** common eider, corticosterone, foraging, migration, reproduction, stable isotopes, temperature, trade‐offs

## Abstract

1. The availability and investment of energy among successive life‐history stages is a key feature of carryover effects. In migratory organisms, examining how both winter and spring experiences carryover to affect breeding activity is difficult due to the challenges in tracking individuals through these periods without impacting their behavior, thereby biasing results.
2. Using common eiders *Somateria mollissima*, we examined whether spring conditions at an Arctic breeding colony (East Bay Island, Nunavut, Canada) can buffer the impacts of winter temperatures on body mass and breeding decisions in birds that winter at different locations (Nuuk and Disko Bay, Greenland, and Newfoundland, Canada; assessed by analyzing stable isotopes of 13‐carbon in winter‐grown claw samples). Specifically, we used path analysis to examine how wintering and spring environmental conditions interact to affect breeding propensity (a key reproductive decision influencing lifetime fitness in female eiders) within the contexts of the timing of colony arrival, pre‐breeding body mass (body condition), and a physiological proxy for foraging effort (baseline corticosterone).
3. We demonstrate that warmer winter temperatures predicted lower body mass at arrival to the nesting colony, whereas warmer spring temperatures predicted earlier arrival dates and higher arrival body mass. Both higher body mass and earlier arrival dates of eider hens increased the probability that birds would initiate laying (i.e., higher breeding propensity). However, variation in baseline corticosterone was not linked to either winter or spring temperatures, and it had no additional downstream effects on breeding propensity.
4. Overall, we demonstrate that favorable pre‐breeding conditions in Arctic‐breeding common eiders can compensate for the impact that unfavorable wintering conditions can have on breeding investment, perhaps due to greater access to foraging areas prior to laying.

## Graphical abstract

The availability and investment of energy among successive life‐history stages is a key feature of carryover effects. Using common eiders, we used path analysis to examine how wintering and spring environmental conditions interact to affect breeding propensity (a key reproductive decision influencing lifetime fitness in female eiders) within the contexts of the timing of colony arrival, prebreeding body mass (body condition), and a physiological proxy for foraging effort (baseline corticosterone). Overall, we demonstrate that in favorable prebreeding conditions, Arctic‐breeding common eiders can compensate for the impact that unfavorable wintering conditions can have on breeding investment, perhaps due to greater access to foraging areas prior to laying.

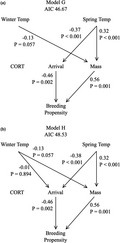

## 1 INTRODUCTION

Across a diversity of species, energetic constraints play important roles in investment decisions at all stages of their annual cycles (Barnes & Partridge, 2003; Coma & Ribes, 2003; Festa‐Bianchet et al., 2019; Lamarre et al., 2017; Schultz et al., 1991). As such, the accumulation and careful management of energetic resources is critical for fuelling transitions between key events or life‐history stages (such as between migration and reproduction) (Alerstam, 2006; Drent et al., 2006; Schultz et al., 1991). Defined as carryover effects, wherein the previous experience of an individual explains its current performance (sensu: O’Connor et al., 2014), these impacts can be driven by multiple factors including the availability of energy and nutrients (Barnes & Partridge, 2003; Harrison et al., 2011; Shertzer & Ellner, 2002; van Noordwijk & de Jong, 1986; Williams, 2012: pp. 247–259). Importantly, since these effects have the potential to impact variation in individual state and performance at subsequent life‐history stages (Shertzer & Ellner, 2002), they also have the potential to impact investment in downstream events such as breeding decisions (Burnett et al., 2017).

Carryover effects are often found in, or exaggerated in, migratory species, since the ability to successfully migrate between wintering and breeding locations is linked to the availability of resources to meet energetic demands on the wintering grounds and during migration (Johnson et al., 2016; Tamisier et al., 1995). It is possible that the extent to which individuals can accumulate and maintain energetic stores during the winter can have significant implications for reproduction, especially with respect to breeding decisions and investment (Crossin, Phillips, et al., 2012; Crossin et al., 2013; Hennin et al., 2018; Martin, 1987; Oosterhuis & Van Dijk, 2002), and breeding success (Burnett et al., 2017; Williams, 2012: pp. 224–225). Indeed, individuals in higher quality wintering habitats often arrive to the breeding site earlier, arrive in higher body mass, lay earlier and have higher reproductive output/success (Drake et al., 2013; Norris et al., 2004; Sorensen et al., 2009).

Variation in habitat quality on the wintering grounds may carry over to impact subsequent reproduction (Imlay et al., 2019; Norris, 2005; Rockwell et al., 2012; Szostek & Becker, 2015). An important mechanism linking wintering habitat quality to reproduction is food availability (Ballesteros et al., 2013; Brown & Sherry, 2006; Shertzer & Ellner, 2002). For some species, food availability can be influenced by variation in abiotic factors such as temperature in wintering environments (Lehikoinen et al., 2006; Williams et al., 2015), while for others breeding parameters can be more heavily influenced by conditions in their immediate, prebreeding environment (Harrison et al., 2011; Van Oudenhove et al., 2014). For instance, among many migratory bird species, the temperatures experienced after arrival on the breeding grounds were more important drivers of lay date than the carryover effects of precipitation (as a proxy for resource abundance and habitat quality) on the wintering grounds (Jean‐Gagnon et al., 2018; Love et al., 2010; Ockendon et al., 2013; Ramírez et al., 2017; Senner et al., 2014). Therefore, despite the negative influences of low quality wintering habitat on important reproductive metrics (e.g., timing of arrival, breeding propensity, reductions in clutch size, and breeding success), favorable conditions during migration and spring arrival on breeding grounds can buffer these negative carryover effects (Bêty et al., 2003; Descamps et al., 2011; Perrins, 1970; Rowe et al., 1994).

Here, we examine the carryover effects of winter temperatures, and how variation in subsequent spring conditions, may influence reproductive parameters of common eider ducks breeding at a colony on East Bay Island in Arctic Canada. Eiders breeding at this colony are an ideal system to test these questions because they migrate thousands of kilometers from either of two primary wintering areas (i.e., either the coast of Western Greenland in Nuuk and Disko Bay, or the coast of Newfoundland, Canada) to breed in the Eastern Canadian Arctic (Mosbech et al., 2006). Importantly, the winter weather driven by the North Atlantic Oscillation (NAO, NOAA, 2018) typically generates opposite environmental conditions in Western Greenland and Newfoundland (Descamps et al., 2010; Mosbech et al., 2006; Steenweg et al., 2017). In positive years, when there are below‐average temperatures in Western Greenland with higher incidences of storms, Newfoundland will experience above‐average temperatures and fewer storms (Descamps et al., 2010; NOAA, 2018). Given that sea ice concertation and temperatures are highly correlated (Comiso, 2002), the differences in NAO and temperatures will also result in a difference in sea ice concentration between these wintering areas (Heide‐Jørgensen et al., 2007). The difference in winter temperatures between these areas has the potential to generate different carryover effects on eider reproduction in the Arctic depending on their wintering location.

Female eider ducks demonstrate a mixed capital‐income reproductive strategy (Sénéchal et al., 2011a). The decision to lay should be strongly influenced by the fat accrued on the wintering grounds as well as the energy gained by foraging near their nesting colony upon spring arrival. Collectively eiders must accrue enough energy to fuel egg formation (Sénéchal et al., 2011a), as well as build enough reserves to successfully complete a 24‐day incubation fast (Bottitta et al., 2003). Therefore, variation in both the resources brought from the wintering grounds, their body condition upon arrival to the breeding grounds, and the ability of hens to quickly gain the additional resources necessary to lay, should predict variation in the decision to breed (Descamps et al., 2011; Hennin et al., 2018; Sénéchal et al., 2011a).

Because eiders are diving sea ducks, which rely on access to open water areas for foraging opportunities, colder winter temperatures can restrict available foraging areas due to increased ice cover (Heide‐Jørgensen et al., 2007; Merkel et al., 2006). We therefore predicted that colder winter temperatures would negatively affect the timing of arrival at the breeding grounds and prelaying body mass (Descamps et al., 2010), and negatively impact breeding propensity. However, if female eiders experienced favorable spring environmental conditions upon arrival at the breeding grounds (i.e., warmer conditions with more ice‐free areas to forage), individuals could buffer against or compensate for winter‐derived energetic shortfalls. Thus, we also predicted that warmer spring temperatures, earlier arrival dates, and higher prebreeding body mass would lead to positive effects on breeding propensity, irrespective of wintering conditions or location.

## 2 MATERIALS AND METHODS

### 2.1 General field methods and sampling

**FIGURE 1:**
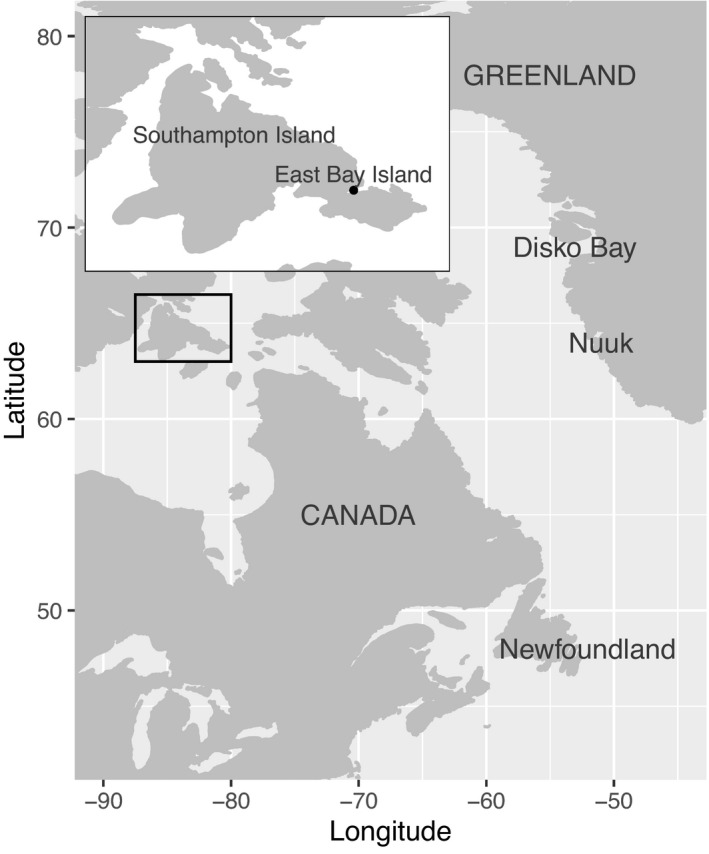
Location of common eider breeding colony on East Bay Island, Nunavut, Canada, and wintering sites at Disko Bay and Nuuk, Greenland, and Newfoundland, Canada

We tested our questions by studying female common eiders nesting at a breeding colony on East Bay Island (Mitivik Island, Nunavut, Canada, 64°02′N, 81°47′W) within the East Bay off of Southampton Island, in the East Bay (Qaqsauqtuuq) Migratory Bird Sanctuary (Figure 1). Common eiders breeding on East Bay Island migrate to their wintering areas (Nuuk and Disko Bay, Greenland and Newfoundland, Canada) after the breeding season (Mosbech et al., 2006). Common eiders remain in wintering areas from December to March and begin their spring migration back to the breeding colony in April, following the receding of the sea ice (Mosbech et al., 2006). Eiders arrive to staging areas near the breeding colony in May and June where they forage to accrue sufficient body mass to last them through the incubation period. Female eiders arrive at the breeding colony late during the pre‐breeding period in mid‐June to early July when they are ready to prospect for nests and lay their eggs (Hennin et al., 2015, 2018; Sénéchal et al., 2011b).

Female common eiders were captured during the pre‐breeding period (mid‐June to early July) from 2014 to 2017 using large flight nets (*n* = 273 individuals). Birds were banded with field‐readable alpha‐numeric plastic bands as well as a metal band from the USGS Bird Banding Laboratory. Each female was also given a combination of uniquely colored and shaped plastic nasal tags threaded through their nares with UV degradable monofilament. This enabled us to identify and monitor individual hens on the colony in June and July, but ensured that all nasal tags fell off prior to fall migration in September. We obtained breeding propensity data for all captured females by surveying the colony twice a day from within observation blinds from mid‐June to mid‐July during the laying period. Individual females were categorized as non‐breeders (*n* = 160) if they did not return to the colony to lay, given the high site fidelity known for this colony (Jean‐Gagnon et al., 2018), and as breeders (*n* = 86) if they were observed incubating eggs (Table 1). Females were considered to be in the laying (*n* = 21) or incubating (*n* = 6) stages if caught once they had already begun laying or known to be incubating, determined through twice daily plot monitoring efforts. These 27 laying and incubating hens were removed from this analysis because their body mass would be influenced by the development and laying of eggs at this time and therefore not an accurate representation of body condition (Descamps et al., 2011) and so our total sample size is *n* = 246 individuals.

**TABLE 1:** Biometric values and sample sizes for breeding and nonbreeding female common eiders captured during the prebreeding season (June and July) in 2014–2017 at East Bay Island. All values are presented ± *SD*

Year	Total *n*			Mean body mass (g)		Mean corticosterone (ng/ml)		Mean Julian arrival date		Mean lay date
	Overall	Nonbreeders	Breeders	Nonbreeders	Breeders	Nonbreeders	Breeders	Nonbreeders	Breeders	
2014	53	29	24	2143 ± 169	2194 ± 168	11.14 ± 9.25	10.35 ± 7.14	176 ± 4	174 ± 5	179 ± 4
2015	63	42	21	1979 ± 218	2165 ± 120	8.17 ± 13.07	12.13 ± 20.15	179 ± 5	178 ± 4	182 ± 4
2016	67	53	14	2130 ± 249	2263 ± 139	10.68 ± 13.37	14.18 ± 11.86	179 ± 5	177 ± 3	181 ± 3
2017	63	36	27	2207 ± 199	2269 ± 202	8.48 ± 15.21	8.96 ± 12.37	173 ± 4	173 ± 3	177 ± 4

All birds were blood sampled from the tarsal vein within 3 minutes of initial capture to obtain baseline physiological metrics (Hennin et al., 2015). We then measured body mass to the nearest 10 g, to assess arrival body condition (Descamps et al., 2011). We then collected the distal 2 mm from the claw of the middle toe on the left foot. Toe clippings were later analyzed for stable isotopes to assign winter location (following, Steenweg et al., 2017). Stable isotopes of 13‐carbon can change with distance to shore or along a latitudinal gradient (Cherel et al., 2008; Steenweg et al., 2017) and are therefore unique to different locations. In common eiders, claws take approximately 120 days to grow from root to tip, so the stable isotope values from the tips of claws obtained on the breeding grounds in June, are associated with the location in which the tissue was grown, that is, where the individual was wintering in January to March (Steenweg et al., 2017). Stable isotopes of 13‐carbon in claws have been successfully used in this colony of common eiders to infer individual wintering areas (Nuuk or Disko Bay, Greenland or Newfoundland, Canada; Steenweg et al., 2017). We used stable isotopes of 13‐carbon from claws collected from eider hens at arrival to their breeding grounds to infer each individual's wintering area.

This noninvasive method, in which we analyzed the stable isotopes of 13‐carbon in claws grown in winter, enabled us to assign the wintering location of migratory females from samples collected at arrival on this breeding colony. This method allowed us to compare wintering and prebreeding spring conditions on reproductive performance without the deployment of bio‐logging devices, which have the potential to bias results through impacts on bird behavior, foraging, reproduction, and survival (Burger & Shaffer, 2008).

### 2.2 Assignment of wintering location and environmental conditions

Briefly, we removed surface oils from claw samples by placing claw subsamples into vials and adding a 2:1 chloroform:methanol solution, vortexing them for 15 s and letting them sit for 24 h. We then centrifuged vials at 10,000 rpm for 10 min and siphoned off the supernatant with a pipette. We rinsed samples with the chloroform:methanol solution and repeated the procedure. Following this, samples were dried in a fume‐hood for 24 h. Subsamples of claws were weighed to 0.30–0.50 mg, and then placed into tin capsules to be analyzed for stable isotopes of carbon (^13^C and ^12^C).

Samples collected from 2014 to 2016 were analyzed at Queen's University, and from 2017 at the Great Lakes Institute of Environmental Research (GLIER) at the University of Windsor. To ensure that these two labs were consistent and comparable in their carbon isotopic measurements, we re‐analyzed 10 randomly selected samples at GLIER that we had previously analyzed at Queen's. These pairs of samples were within 0.4 ± 0.8 (*SD*) of each other, indicating each sample was sufficiently homogenous and the results of the two labs were indeed comparable. All stable isotope results are reported within accuracy of 0.1‰ based on analyses of the international standard Vienna Pee Dee Belemnite and in‐house keratin (COW1: −13.17‰ ± 0.21, UC1: −25.7‰ ± 0.14) run alternately every five samples. To assess accuracy of our measurements, duplicates were run every nine samples with an accuracy of 0.2‰. All ^13^C/^12^C are reported in delta notation (δ) in parts per mil (‰).

To establish the general wintering conditions of each individual eider, we generated data for winter conditions for each year by averaging temperatures from January to March in each of the three common eider wintering areas: Nuuk, Greenland; Disko Bay, Greenland (Cappelen, 2018); and Cartwright in Newfoundland, Canada (Environment & Climate Change Canada, 2018). We generated data on spring conditions at East Bay by averaging the temperature for May from the nearest weather station located at Coral Harbour, Southampton Island, Nunavut, Canada located 45 km from the breeding colony (Environment & Climate Change Canada, 2018) (Table 2).

**TABLE 2:** Temperatures at wintering sites in Newfoundland, Canada, and Nuuk and Disko Bay, Greenland, and at the breeding site at East Bay, Southampton Island, Nunavut in spring for the years 2014–2017

Year	Winter temperature (℃)			Spring temperature (℃)
	Newfoundland, Canada (*n*)	Nuuk, Greenland (*n*)	Disko Bay, Greenland (*n*)	Southampton Island, Nunavut (*n*)
2014	−13.6 (9)	−7.5 (36)	−10.4 (8)	−3.0 (53)
2015	−16.5 (6)	−11.3 (55)	−17.2 (2)	−7.7 (63)
2016	−13.3 (30)	−5.3 (37)	na	−4.9 (67)
2017	−12.2 (17)	−7.4 (46)	na	−4.2 (63)

Note

In years where eiders did not winter in the area, temperatures were not applicable (na). Sample size of female common eiders arriving from each wintering site in each year is denoted by n.

### 2.3 Physiological indicator of foraging effort ‐ baseline corticosterone

We included baseline corticosterone (CORT) measured from plasma samples collected at capture in our models given that elevations in baseline CORT have been linked to increases in foraging behaviors, mass gain, and energetic demand during the prebreeding period (Angelier et al., 2007; Crossin, Trathan, et al., 2012; Hennin, Bêty, et al., 2016; Holberton, 1999; Love et al., 2014). Baseline CORT was measured using an enzyme immunoassay (EIA; Assay Designs, Ann Arbor, MI, USA) previously validated in common eiders breeding at East Bay (Hennin et al., 2015). All samples were run in triplicate at 1:20 dilution with 1.5% steroid displacement buffer by volume, in random order and in a 96‐well plate. Each plate included a control of laying hen plasma (Sigma–Aldrich Canada, Oakville, Ontario, Canada) and a kit‐provided, serially diluted standard curve (200,000 pg/ml). Plates were read at 405 nM. The inter‐ and intraplate coefficients of variation were 9.96% and 19.26%, respectively.

### 2.4 Data analysis

To determine wintering sites of individual arriving common eiders, we used a k‐means cluster analysis of the stable isotope data derived from claws of common eiders arriving to the breeding colony (as per Steenweg et al., 2017). K‐means cluster analysis is a centroid‐based clustering method where the centroids are the iteratively calculated centers of the clusters and where *k* is the number of clusters (Tan et al., 2006; in this case *k* = 3, one for each wintering site). In this method, it is possible to predetermine the starting centroids for the clusters. Each of the remaining points is assigned to a cluster in a way which minimizes the sum of squared error of each centroid (Tan et al., 2006). The *k*‐means cluster analysis was informed by using the previously published starting centroids calculated from the means of the stable isotope data obtained from common eiders on their wintering sites for this breeding colony (Nuuk: −14.92‰, Disko Bay: −18.12‰ and Newfoundland: −20.55‰; Steenweg et al., 2017).

We used piecewise structural equation modeling (R package piecewiseSEM Version 2.0.1; Lefcheck et al., 2019) to test whether environmental variables (winter and/or spring temperatures) directly predicted our response variable (breeding propensity), or whether these relationships were mediated through effects on other variables (i.e., arrival date, CORT, and/or body mass) (Lefcheck, 2016; Shipley, 2013). This approach allowed us to determine direct and indirect correlations between spring and winter conditions, arrival date, CORT, body mass, and how these together influenced breeding propensity (Hennin et al., 2018; Lefcheck, 2016).

**FIGURE 2:**
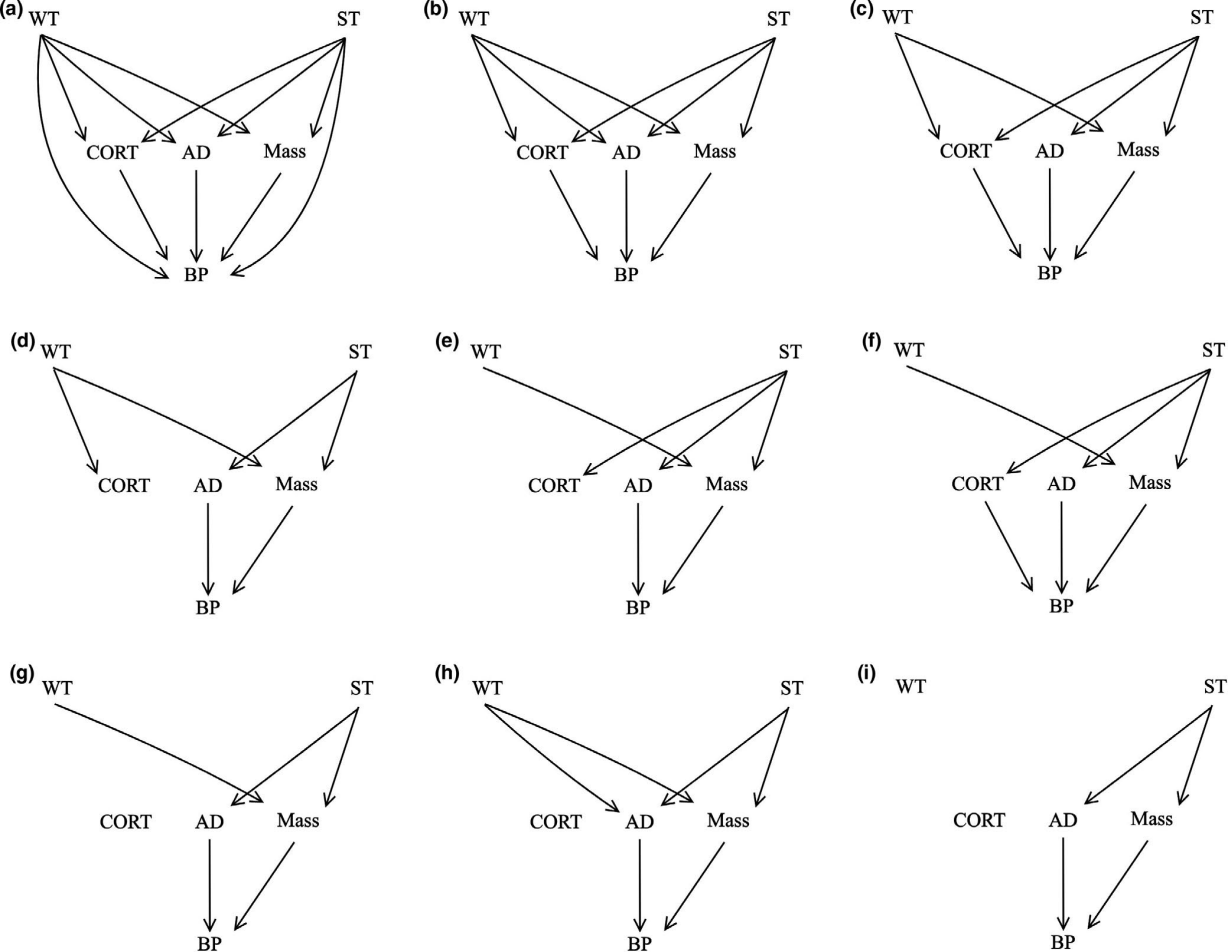
Diagrams of the 9 hypothesized, biologically feasible path models linking environmental conditions to breeding propensity in female common eiders. The variables included in the models are winter temperature (WT), spring temperature (ST), baseline corticosterone (CORT), arrival date (AD), body condition (Mass), and breeding propensity (BP)

We constructed nine separate conceptual path models, each with biologically feasible linkages among the variables (Figure 2). These models were then converted to a set of conditional dependencies, which were then analyzed as generalized linear mixed models with sample number as a random intercept (to account for shared variance in sampling order). We used Gaussian models with identity function (normally distributed data for spring and winter temperatures, arrival date, baseline CORT, and body mass) and standardized these data to allow effects to be compared across the multiple responses and a binomial model with logit function (binomial data; breeding propensity) (Lefcheck, 2020). We ranked each model with Akaike Information Criterion (AIC) within *piecewiseSEM* to assess the strongest candidate models. We calculated path coefficients and *p*‐values for these top models. Given that it may be difficult to tease apart whether the potential effects of winter are due to location‐specific temperatures or other factors that are specific to the wintering area (e.g., availability of preferred food sources; Goudie & Ankney, 1986; Merkel et al., 2007), we subsequently used a linear model mirroring that of the best ranked path analysis to test for effects of winter location on arrival body mass. We also included spring temperature to account for effects of spring.

## 3 RESULTS

**FIGURE 3:**
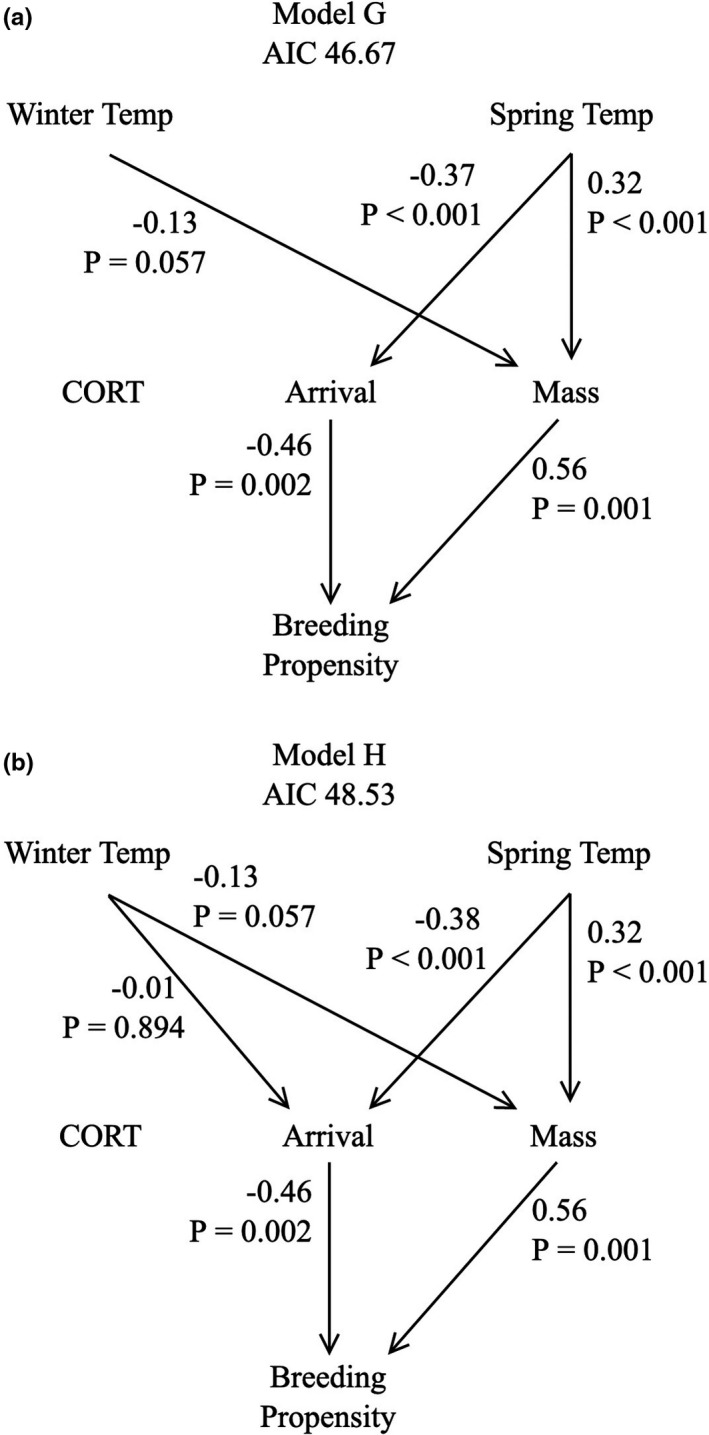
Diagrams of the top two ranked paths as determined by AIC rank linking spring and winter temperatures to breeding propensity. Standardized path coefficients and *p*‐values for each relationship are reported next to its corresponding arrow

Analyses generated two competitive models (Models G and H; Figure 3) within two ΔAIC values of each other (Model G: AIC = 46.67, Fisher's C statistic = 20.67, *p* = .11, *df* = 14; Model H: AIC = 48.53, Fisher's C statistic = 20.53, *p* = .06, *df* = 12; Table 3). The two highest ranked models had similar structure, including negative linkages between winter temperatures and body mass, as well as significant positive linkages between spring conditions and both earlier arrival date and heavier body mass.

**TABLE 3:** Comparisons of the path models linking the effects of winter and spring temperatures to circulating baseline CORT, arrival date, body mass, and breeding propensity in female common eiders captured at arrival during the pre‐breeding season at East Bay Island

Model Rank	Model	AIC	ΔAIC	Fisher's C	*p*	*df*
1	G	46.67	0	20.67	.11	14
2	H	48.53	1.86	20.53	.06	12
3	I	51.29	4.62	27.29	.04	16
4	D	56.02	9.35	22.02	.14	16
5	C	56.75	10.08	18.75	.09	12
6	F	58.32	11.65	22.32	.07	14
7	E	58.45	11.78	24.48	.08	16
8	B	58.53	11.86	18.53	.05	10
9	A	59.56	12.89	15.56	.02	6

Note

The path structure of these models is included in Figure 2 according to their model letter. This analysis includes pre‐recruiting, rapid follicle growth and nonbreeding birds. Incubating and laying hens were excluded as they did not truly represent “arriving” birds.

Contrary to our predictions, colder winter temperatures predicted higher body mass (standardized path coefficient = −0.13, *p* = .057), with no significant effects on arrival dates (Figure 3a, model G). Overall, warmer spring temperatures on the breeding grounds predicted earlier arrival dates (standardized path coefficient = −0.37, *p* < .001) and higher body mass (standardized path coefficient = 0.32, *p* < .001). Birds with higher body mass were more likely to breed (standardized path coefficient = 0.56, *p* = .001) as were those that arrived at the colony earliest (i.e., predicting higher breeding propensity; standardized path coefficient = −0.46, *p* = .002). Neither of the top models included direct effects of either spring or winter conditions on breeding propensity, these effects were mediated through body mass and arrival date. Neither of the top models included links between spring or winter conditions on CORT, nor CORT on breeding propensity. Our follow‐up analyses testing for effects of wintering location on arrival body mass found that individuals arriving from Nuuk, Greenland had significantly lower body mass compared to those arriving from Newfoundland (estimate = −0.35, *p* = .01;) and arrival body mass was significantly and positively associated with spring temperatures (Estimate = 0.24, *p* < .001 model estimates: *F*_(2,242)_ = 8.57, *p* < .01, *R* ^2^ = .08, Table 4).

**TABLE 4:** Summary of parameter estimates of fixed effects from linear models of analyses investigating effects of winter location (relative to Newfoundland) and spring temperature on arrival body mass

Response	Variable	Estimate	± *SE*	*t*	*p*
Mass	Intercept	0.25	0.12	2.08	.04
	Disko Bay, Greenland	−0.14	0.33	−0.43	.67
	Nuuk, Greenland	−0.35	0.14	−2.46	.01
	Spring temperature	0.24	0.06	3.98	<.001

## 4 DISCUSSION

### 4.1 Impacts of spring conditions on mass‐dependent breeding decisions

We investigated a multiyear data set to examine the relative contributions of both winter and spring environmental conditions on important arrival traits and a key reproductive decision to assess the relative impacts of seasonal carryover effects in female common eiders breeding in the Arctic. We found that during years with warm spring conditions occurring near the nesting colony, female eiders arrived at the colony earlier and in better body condition (i.e., higher body mass). In years with relatively colder winter temperatures, eiders also arrived with higher body mass. It is noteworthy that the overall influence of spring conditions had a 2–3 times greater impact on reproductive metrics than did winter conditions (Figure 2). Overall, our results indicate that females that arrive to breeding areas under favorable spring environmental conditions are better able to mitigate negative carryover effects of challenging winters.

Previous research of common eiders in the Eastern Canadian Arctic has demonstrated that harsh conditions on their wintering grounds negatively impact arrival body mass in females (Descamps et al., 2010), and that arrival body mass is a strong predictor of the timing of reproduction, clutch size, and hatching success (Descamps et al., 2010; Hennin, Bêty, et al., 2016; Hennin et al., 2018). Additionally, our path analysis also suggests that females can overcome some of the negative impacts of wintering conditions to invest in reproduction. We found that warmer spring temperatures resulted in advanced dates of arrival and increased body mass, both of which increased eider duck breeding propensity. Female body mass was a key intrinsic variable linking extrinsic environmental conditions (temperatures) to breeding propensity. This is consistent with other studies demonstrating the key role body mass plays in mediating reproduction in common eiders (Descamps et al., 2010; Hennin, Bêty, et al., 2016; Hennin et al., 2018).

It is likely that the strong relationship between spring temperatures on the breeding grounds and the subsequent positive effects on reproductive decisions is mediated by local sea ice conditions on the breeding grounds that strongly impact regional foraging conditions of this marine bird (Jean‐Gagnon et al., 2018). In years with warmer spring temperatures, there is more available open water and eiders lay earlier (Jean‐Gagnon et al., 2018), presumably via more extensive foraging opportunities that enable females to quickly accrue the fat reserves necessary to support clutch formation and egg laying. Our results also help to mechanistically explain previous findings at this colony linking warmer spring conditions to earlier breeding phenology and breeding success (Love et al., 2010), positive links between elevated prebreeding fattening rates and earlier lay dates (Hennin, Bêty, et al., 2016), and the importance of elevated body mass in driving the seasonal decline in clutch size in common eiders (Descamps et al., 2011).

### 4.2 Effects of corticosterone on mass‐dependent breeding decisions

Remarkably, variation in baseline CORT did not emerge as a significant predictor of breeding propensity. We had anticipated that variation in baseline CORT would be a significant physiological mediator linking winter and/or spring temperatures to breeding propensity, via its role as a metabolic regulator of daily activity, foraging behavior, and body mass gain (Crossin, Trathan, et al., 2012; Hennin et al., 2016). Despite the lack of an apparent significant impact in this study, baseline CORT has been shown to be an important regulator in the energetics of prelaying eiders (Hennin et al., 2015; Hennin, 2016). Female common eiders have been shown to increase baseline CORT secretion as they transition from the prerecruiting to the rapid follicle growth period (Hennin et al., 2015). Manipulation experiments in captive diving sea ducks have shown that experimentally elevated baseline CORT results in an increase in body fat (Hennin, Wells‐berlin, et al., 2016), and experimentally elevated baseline CORT in free‐living eiders advanced lay dates and increased breeding success (Hennin, 2016). In our analysis we needed to include both breeding and nonbreeding birds to examine impacts of carryover effects on the probability of breeding within a given year. It is possible that the role of baseline CORT as physiological/energetic mediator may have been diminished by including nonbreeding birds, since nonbreeders may have little to no need to meet the same mass thresholds for breeding.

### 4.3 Effects of winter conditions and location on breeding decisions

Winter temperatures had a nearly significant negative relationship with body mass in female eiders. However, our results also indicate that the effects of winter on arrival body mass may not be due to temperatures per se, but rather wintering location; individuals arriving from Nuuk, Greenland had a lower body mass than those arriving from Newfoundland. There are two primary reasons birds arrived from Newfoundland with higher body mass compared to those arriving from Nuuk, Greenland. Eider diet in Newfoundland contains a higher proportion of mussels (Newfoundland: Goudie & Ankney, 1986; Greenland: Merkel et al., 2007), which is a preferred diet item due to their higher energy content (Goudie & Ankney, 1986; Guillemette, 1998; Larsen & Guillemette, 2000; Merkel et al., 2007). Secondly, wintering sites in Newfoundland are closer to the eventual breeding colony than Western Greenland (Mosbech et al., 2006). Since the energetic costs of flight in common eiders are high (Pelletier et al., 2008), eiders wintering in different locations may face different costs of migration, have differing quality of prey sources at those wintering sites to fuel migration, and likely a differing ability to carry fat stores with them from the breeding grounds, impacting arrival mass. Ultimately, although our study is an initial step toward assessing their potential for carryover effects in common eiders, the effects of wintering location are likely very complex.

### 4.4 Mechanisms driving variation in carryover effects

Our findings suggest that female common eiders are able to buffer winter carryover effects if they encounter favorable (i.e., warm) spring conditions during their prebreeding period, which can last upward of one month after arrival on the breeding grounds (Mosbech et al., 2006). In fact, our findings suggest that the positive effect of spring temperatures on arrival body mass is more than twice that of favorable winter conditions. During the spring, birds may be able to compensate for the energetic shortfalls resulting from conditions on their wintering grounds (Merkel et al., 2006; but see Jamieson et al., 2005), as well as the energetic costs stemming from spring migration. Wintering conditions may prevent individuals from forming pairs prior to arrival and recent data suggest that some eiders also use this arrival spring period for pair formation (Steenweg et al., 2019). These results underscore the importance of early timing of arrival to the breeding grounds during the prebreeding period for proximate energy gain, potentially finding a mate, investment in breeding, and ultimately for fitness benefits.

Winter carryover effects often occur or have the strongest effects in species with a short prebreeding period (i.e., interval between arrival and breeding) and can be further impacted by breeding strategies (i.e., more capital or income based resource use; Meijer & Drent, 1999). The prelaying period is important for gaining sufficient mass to fuel egg development across multiple species (e.g., macaroni penguins *Eudyptes chrysolophus*; Crossin et al., 2010, and white‐winged scoters *Melanitta fusca*; Gurney et al., 2014). Overall, common eiders have a relatively long prelaying period (up to 20 days; Hennin et al., 2015) and therefore should have the flexibility to overcome potential carryover effects. In support of this, we found that female eiders are indeed capable of overcoming wintering carryover effects. We recognize that there are likely individual‐based differences in the ability to compensate for the effects of challenging wintering conditions including wintering location (Descamps et al., 2010), foraging and assimilation ability (Bond & Esler, 2006; Heath et al., 2010; Rigou & Guillemette, 2010), and physiological fattening rates (Hennin et al., 2018).

Carryover effects may also be demonstrated or mitigated through differential reliance on more capital (endogenous) or income (exogenous) based energetic reserves. Income breeders are largely affected by prey source availability on the breeding grounds and as such, exhibit little to no winter carryover effects (Guillemain et al., 2008; Senner et al., 2014). However, common eiders use a combination of capital and income based resources to fuel egg growth (Clausen et al., 2015; Descamps et al., 2011; Provencher et al., 2016; Sénéchal et al., 2011b); the relative contribution of which may vary depend on wintering conditions and its effects on arrival body mass (Descamps et al., 2010). Specifically, harsher winter conditions likely make it challenging for females to maintain a high amount of fat stores to bring over to the breeding grounds, and under these circumstances, they must rely more on income‐based resources just prior to breeding (Sénéchal et al., 2011b). The opposing wintering conditions that females at this colony are exposed to at their different wintering locations (Steenweg et al., 2017) can indeed impact their arrival mass with downstream consequences for reproductive decisions (Descamps et al., 2011; Hennin, Bêty, et al., 2016; Hennin et al., 2018; this study). The consistency and strength of the relationship between spring temperature and arrival body mass in our competitive models indicate that spring conditions (likely mediating foraging opportunities and the accumulation of capital stores) are currently the most critical extrinsic factor affecting the ability of females to invest in reproduction in a given year (Descamps et al., 2011; Hennin, Bêty, et al., 2016; Hennin et al., 2018), regardless of the effects of winter conditions.

For long‐lived marine birds such as the common eider, the decision to breed in any given year plays a significant role in contributing to lifetime reproduction. Nonetheless, skipping or deferring breeding may be an appropriate tactic to deal with variable, and increasingly unpredictable environmental conditions (Legagneux et al., 2016; Öst et al., 2018; Shaw & Levin, 2013), such as unfavorable spring conditions examined here. Skipping reproduction can result in increased chances of subsequent survival (i.e., trade‐off between current and future reproduction; Shoji et al., 2015), and even increase the likelihood of breeding in the following year (Legagneux et al., 2016; Jean‐Gagnon et al., 2018; see also Catry et al., 2013, in shearwaters; Crossin et al., 2017, in albatrosses). In doing so, a bird could capitalize on years with more agreeable conditions and increase their lifetime reproductive output (Coulson, 1984; Reed et al., 2015). In common eiders, although spring conditions could lead to deferred or skipped breeding for some individuals (particularly following poor winters and unfavorable spring conditions at arrival), it is possible that this reproductive deferral and investment in long‐term self‐maintenance, may carryover to increase a female common eider's mass the following winter and the likelihood of breeding in the subsequent year. Therefore, although challenging, future studies that are able to test these questions and relationships by monitoring individuals across multiple seasons and years would help to elucidate individual‐based strategies for mitigating trade‐offs and carryover effects.

## CONFLICT OF INTEREST

The authors do not have any conflicts of interest.

## AUTHOR CONTRIBUTIONS

**Rolanda J. Steenweg:** Conceptualization (equal); Data curation (lead); Formal analysis (lead); Methodology (lead); Writing – original draft (lead). **Glenn T. Crossin:** Conceptualization (equal); Funding acquisition (equal); Resources (equal); Supervision (equal); Writing – review & editing (equal). **Holly L. Hennin:** Conceptualization (equal); Data curation (equal); Writing – review & editing (equal). **Hugh Grant Gilchrist:** Conceptualization (supporting); Data curation (equal); Project administration (lead); Resources (equal); Writing – review & editing (equal). **Oliver P. Love:** Conceptualization (equal); Investigation (equal); Methodology (equal); Supervision (equal); Writing – review & editing (equal).

## Data Availability

The data that support the findings of this study are openly available in Dryad at https://doi.org/10.5061/dryad.ttdz08m0x.
